# Prostate Cancer: From Molecular Imaging to Immunological and Target Therapies

**DOI:** 10.3390/biomedicines11041176

**Published:** 2023-04-14

**Authors:** Luca Filippi, Agostino Chiaravalloti

**Affiliations:** 1 Nuclear Medicine Unit, “Santa Maria Goretti” Hospital, Via Antonio Canova, 04100 Latina, Italy; 2 Department of Biomedicine and Prevention, University Tor Vergata, Viale Oxford 81, 00133 Rome, Italy; agostino.chiaravalloti@uniroma2.it; 3 IRCCS Neuromed, 86077 Pozzilli, Italy

Prostate cancer (PCa) is one of the most common malignancies and a leading cause of cancer-related deaths, affecting a million people worldwide with a particularly high burden in countries with a low human development index [1]. Although population screening by prostate-specific antigen (PSA) testing has favorably impacted PCa mortality, this approach still remains controversial due to its high sensitivity but relatively low specificity. Therefore, there is an unmet need for effective laboratory and imaging biomarkers in all phases of PCa’s natural history, from diagnosis up until its ultimate switch into the more aggressive and androgen-independent form (metastatic castration-resistant prostate cancer/mCRPCa).

Hence, the Special Issue “Prostate Cancer: from Molecular Imaging to Immunological and Target Therapies” was conceived as an opportunity to disseminate the current research on this cutting-edge topic, with a particular emphasis on recently implemented metabolic and molecular imaging probes in the field. For example, Urso et al. investigated the impact of positron emission computed tomography (PET/CT) using ^18^F-choline, a surrogate biomarker of cell membrane biosynthesis, on the initial clinical management of PCa patients according to a multidisciplinary approach [2]. The enrolled patients were split into three distinct groups based on the type of imaging they received: A) conventional radiological imaging (MRI or CT); B) conventional imaging plus ^18^F-choline PET/CT; C) only ^18^F-choline PET/CT. The authors found that ^18^F-choline PET/CT was more frequently employed in patients with higher Gleason or International Society of Urological Pathology (ISUP) scores; in addition, the authors found that the execution of ^18^F-choline PET/CT influenced the clinical decisions in 14.3% of patients in group B and correlated with a longer time interval before relapse. Of note, metabolically active tumor burden at initial staging (i.e., total lesion choline kinase activity/TLCKA) and mean standardized uptake value (SUVmean) were able to discriminate patients who experienced tumor recurrence from those who did not relapse.

PET/CT using anti-1-amino-3-[18F]-flurocyclobutane-1-carboxylic acid (^18^F-FACBC) has been introduced into clinical practice for the imaging of PCa patients showing biochemical recurrence (BCR) after radical treatment (radiation therapy/surgery). However, the diagnostic performance of ^18^F-FACBC PET/CT in subjects with low or very low PSA values and the eventual impact of this imaging modality on clinical management have yet to be fully elucidated. In a bicentric retrospective study by Filippi et al., a cohort of 81 PCa patients with BCR after radical therapy underwent ^18^F-FACBC PET/CT [3]. The detection rate in the whole cohort was 76.9% and, when stratified by PSA values, diagnostic performance of ^18^F-FACBC remained relevant for low and very low levels (i.e., 0.58–0.99 and 0.2–0.57 ng/mL, respectively). The most frequent sites of relapse were in prostate bed and pelvic lymph nodes, easily identified thanks to the lack of ^18^F-FACBC physiological accumulation within the bladder. Notably, PET/CT significantly influenced patients’ management in almost half of the cases (40.7%), leading to a major change in the planned treatment (e.g., from androgen deprivation therapy into targeted radiation therapy) in 30 subjects.

Prostate-specific membrane antigen (PSMA) has recently emerged as a promising target for the approaches to PCa based on the synergism between diagnosis and therapy, also known as “theranostics”. The recently published pro-PSMA trial has shown the superiority of PET/CT with ^68^Ga-PSMA-11 over the conventional cross-sectional imaging (CT) and bone scan for the staging of high-risk PCa [4]. However, the role of PSMA PET/CT in low-risk PCa patients still remains to be addressed. From this perspective, Maserumule and coworkers investigated the potential of ^68^Ga-PSMA-11 PET/CT in black South African (BSA) and white South African (WSA) men with ISUP grade 1 (Gleason 3 + 3) and 2 (Gleason 3 + 4) PCa at initial staging [5]. The authors found a correlation between PSA levels and primary tumor and whole-body PSMA-tumor volume (PSMA-TV), with higher values in BSA than in WSA males. Of note, evidence of seminal vesicle invasion at PSMA examination and black ethnicity predicted metastases.

Among the papers included in the Special Issue, two were focused on the radioligand therapy (RLT) with PSMA ligands. Although PSMA RLT with radionuclides emitting beta or alpha particles has been gaining an ever-increasing interest among the scientific community, some issues still remain to be addressed [6]. In particular, although the detection of PSMA-avid lesions on PSMA PET is an essential prerequisite to be enrolled, not all the patients respond to RLT. In this regard, the retrospective study carried out by van der Sar et al. could outline the identikit of the “ideal patient” for PSMA RLT [7]. In the 32 mCRPCa patients submitted to baseline ^68^Ga-PSMA-11 PET/CT before RLT with ^177^Lu-PSMA-617, the authors investigated the prognostic value of baseline PET-derived parameters on imaging response assessed by PSMA-PET after two cycles. In the lesion-level analysis, a correlation was found between SUVpeak/max and response, regardless of the anatomical location of the metastatic site (lymph node, viscera or bone). In the patient-level analysis, SUVpeak value of the most avid metastasis was associated with PET response. Therefore, the lesions’ baseline SUV values, reflecting intralesional PSMA availability, are a potential prognostic factor for RLT outcome. Another relevant topic, in the perspective of the widespread implementation of PSMA RLT, is represented by our increased understanding of the pharmacokinetics (PK) and pharmacodynamics (PD) of the various PSMA ligands. In this regard, van der Gaag and colleagues provided a comprehensive overview of the existing scientific literature concerning this cutting-edge topic, shedding light on the synergism between concomitant therapeutic regimens (e.g., androgen-receptor signaling inhibitors/ARSI) and PSMA RLT, thanks to ARSI-induced up-regulation of PSMA expression on PCa cell membranes [8]. In addition, other relevant issues, such as PSMA dosing, the optimal interval-time between the various cycles and the more frequent adverse events associated with RLT, are covered.

In conclusion, the articles published in this Special Issue provides a snapshot of the state of the art concerning the employment of metabolic and molecular imaging probes in the different phases of PCa’s natural history, with a particular attention to the potential of each radiopharmaceutical to achieve a patient-tailored therapeutic approach.
